# Mapping tonotopic organization in human temporal cortex: representational similarity analysis in EMEG source space

**DOI:** 10.3389/fnins.2014.00368

**Published:** 2014-11-12

**Authors:** Li Su, Isma Zulfiqar, Fawad Jamshed, Elisabeth Fonteneau, William Marslen-Wilson

**Affiliations:** ^1^Department of Psychiatry, University of CambridgeCambridge, UK; ^2^Department of Psychology, University of CambridgeCambridge, UK; ^3^MRC Cognition and Brain Sciences UnitCambridge, UK

**Keywords:** MEG, tonotopy, auditory cortex, spatiotemporal searchlight, RSA

## Abstract

A wide variety of evidence, from neurophysiology, neuroanatomy, and imaging studies in humans and animals, suggests that human auditory cortex is in part tonotopically organized. Here we present a new means of resolving this spatial organization using a combination of non-invasive observables (EEG, MEG, and MRI), model-based estimates of spectrotemporal patterns of neural activation, and multivariate pattern analysis. The method exploits both the fine-grained temporal patterning of auditory cortical responses and the millisecond scale temporal resolution of EEG and MEG. Participants listened to 400 English words while MEG and scalp EEG were measured simultaneously. We estimated the location of cortical sources using the MRI anatomically constrained minimum norm estimate (MNE) procedure. We then combined a form of multivariate pattern analysis (representational similarity analysis) with a spatiotemporal searchlight approach to successfully decode information about patterns of neuronal frequency preference and selectivity in bilateral superior temporal cortex. Observed frequency preferences in and around Heschl's gyrus matched current proposals for the organization of tonotopic gradients in primary acoustic cortex, while the distribution of narrow frequency selectivity similarly matched results from the fMRI literature. The spatial maps generated by this novel combination of techniques seem comparable to those that have emerged from fMRI or ECOG studies, and a considerable advance over earlier MEG results.

## Introduction

For most sensory systems, the spatial organization of cortical neuronal responses resembles that of the sensory surfaces, for example, retinotopy in vision, cochleotopy in audition, and somatotopy in the cutaneous senses. Although tonotopy has been found in non-human primates in the cochlea, the auditory brainstem and auditory cortex (Merzenich and Brugge, 1973; Gross et al., 1974; Ryan and Miller, 1978; Zwiers et al., 2004), it was historically difficult to observe this in humans before the advent of non-invasive neuroimaging methods (Ojemann, 1983; Howard et al., 1996). Here, we argue that the methods used to date are limited in their capacity to capture the rich temporal dynamics intrinsic to auditory processing (Gutschalk et al., 2004), and that improved methods are needed to map the neural substrates of auditory processing, in real time and non-invasively, in the human brain.

In recent decades, non-invasive methods, primarily MEG and EEG, have played an increasingly important role in neuroimaging investigations of auditory processes ranging from elementary auditory perception to complex speech processing (e.g., Luo and Poeppel, 2007). A pioneering neuromagnetic study by Romani et al. (1982), for example, used a single gradiometer device to provide evidence suggesting tonotopic organization in human auditory cortex. Most subsequent MEG studies used equivalent current dipole (ECD) methods to model the MEG sources for the N100 component (Sarvas, 1987), typically finding a gradient from high to low frequency in auditory cortex. Specifically, dipoles that correspond to high frequency inputs are located in deeper parts of the superior temporal plane, while low frequency dipoles are located in more superficial parts of the superior temporal plane (Romani et al., 1982; Pantev et al., 1996; Huotilainen et al., 1998; Cansino et al., 2003). However, it is now recognized that the assumptions made by such ECD methods are not empirically defensible (Lutkenhoner et al., 2003). In particular, when multiple sources are likely to exist in auditory cortex, it is unrealistic to assume that there is a single dipole in this region and results in unreliable conclusions about the organization of the auditory system (Lutkenhoner et al., 2003). In addition, the exclusive focus on the N100 component in many earlier MEG studies militated against exploring frequency responses in other time windows—although other auditory components have been examined in some studies (e.g., Pantev et al., 1995).

It is only in the last decade, when high field (≥3 T) fMRI began to be routinely used to explore the human brain, that more detailed tonotopic maps of human auditory cortex have emerged. A series of important studies have identified at least two tonotopic maps in human auditory cortex composing of multiple gradients from high to low frequency specific brain regions (e.g., Formisano et al., 2003; Dick et al., 2012; Moerel et al., 2012, 2013). These fMRI studies have superseded the earlier MEG research by virtue of their superior spatial resolution. Nonetheless, BOLD fMRI is driven by the slow processes of blood flow and only indirectly reflects the underlying neural processes that generate changes in the BOLD response. The intrinsically sluggish and indirect nature of these measures makes them unable to capture the millisecond-by-millisecond temporal dynamics of neural processing, which is a core property of the auditory system (e.g., Gutschalk et al., 2004).

To overcome these and other shortfalls of the existing approaches used to non-invasively investigate human auditory cortex, we have combined a multivariate pattern analysis method, called Spatiotemporal Searchlight Representational Similarity Analysis (ssRSA), with a sliding time window approach to decode information about frequency preference and selectivity directly from the dynamic neural activity of the brain as reconstructed in combined MEG and EEG (EMEG) source space (Su et al., 2012). This method is an extension of the fMRI RSA (Kriegeskorte et al., 2006) to time resolved imaging modalities. All analyses in this paper were carried out using the Matlab Toolbox for RSA (Nili et al., 2014) and its MEG/EEG extension (http://www.mrc-cbu.cam.ac.uk/methods-and-resources/toolboxes/).

The reason for using time resolved imaging modalities is that the auditory cortex has to process an auditory input with rich, millisecond-by-millisecond temporal dynamics. Using this new approach, we focus on a key temporal property of auditory inputs such as speech—the complex and communicatively critical variations in frequency in the speech input over time—and on the cortical structures that dynamically process and represent these variations, tonotopically or otherwise. We map out both frequency preference and selectivity in bilateral superior temporal areas using combined MEG, EEG, and MRI data, where *frequency preference* refers to the dominant frequency range that a specific brain region may encode, and *frequency selectivity* refers to how broad or narrow is the frequency response of this same region. These two metrics tap into fundamental and important aspects of auditory processing.

The core procedure in ssRSA is the computation of similarity structures that express the dynamic patterns of neural activation at specific points in space and time. This similarity structure is encoded in a representational dissimilarity matrix (RDM), where each cell in the RDM is the correlation distance between the neural activation patterns elicited by pairs of experimental conditions (in this context, auditory stimuli). These *brain data RDMs*, reflecting the pattern of brain activity within the spatiotemporal window defined by the searchlight procedure (Kriegeskorte et al., 2006, 2008a), are then related to *model RDMs*, which express specific theoretical hypotheses about the properties of this activity. In the current study, exploring the frequency preferences and selectivity of auditory cortex, the model RDMs capture the similarity between each stimulus at each frequency band, derived from a computational model of the early stages of auditory processing (Patterson, 1987). This cross-correlational procedure makes it possible to relate low-level neural patterns directly to abstract higher-level functional hypotheses about the organization of the auditory cortex.

To illustrate and investigate the flexibility and the potential power of this ssRSA approach, and following the lead of Moerel et al. (2012, 2013), we use tokens of natural speech (single words) to probe the activity in human auditory cortex. The RSA approach does not require a stimulus set to be pre-structured in advance to test a set of hypotheses. Natural speech intrinsically contains variations in its frequency properties, and if these can be extracted and identified using computational modeling methods, then these more ecologically valid stimuli can form the basis for a set of model RDMs exploring the properties of the neural response to these variations. Although it will also be desirable to use the RSA approach with stimuli such as pure tones, the results of Moerel et al. (2012, 2013), using high-field fMRI, suggest a good concordance between the results for pure tones and the results for natural sounds. This is a comparison that we hope to pursue in the ssRSA/EMEG environment in future research.

In terms of neuroimaging methods, we record EEG simultaneously with MEG because the use of combined MEG and EEG delivers better source localisation than either of these modalities alone. This is because of their complementary sensitivity to neural generators at different orientations and depths (Sharon et al., 2007; Molins et al., 2008; Goldenholz et al., 2009; Henson et al., 2009; Hauk and Stenroos, 2014). The combination of MEG and EEG with neuroanatomical constraints from structural MR for each participant leads to still better source reconstruction results. We combine these three sources of constraint using well established minimum norm estimation techniques (Hämäläinen and Ilmoniemi, 1994; Gramfort et al., 2014), rather than the problematic ECD approach.

The ssRSA method, finally, not only provides a dynamic perspective on the functional organization of human auditory cortex, but also has the potential to relate directly the functional properties of human auditory cortex to neurophysiological evidence from behaving animals, as Kriegeskorte et al. (2008b) have already demonstrated in the visual domain.

## Methods

### Participants, materials, and procedures

Seventeen right-handed native speakers of British English (6 males, mean age = 25 years, range = 19–35, with self-reported normal hearing and no history of hearing problems) were recruited for the study. All gave informed consent and were paid for their participation. The study was approved by the Peterborough and Fenland Ethical Committee (UK).

The study used 400 English verbs and nouns (e.g., *talk, claim*) some of which had past tense inflections (e.g., *arrived, jumped*). These materials were prepared for another experiment, and we assume (a) that their linguistic properties were independent of the basic auditory parameters being examined here and (b) that they provide a reasonably extensive and random sample of naturally occurring frequency variation in human speech. All analyses conducted here were restricted to the first 200 ms of each word. The stimuli were recorded in a sound-attenuated room by a female native speaker of British English onto a DAT recorder, digitized at a sampling rate of 22 kHz with 16-bit conversion, and stored as separate files using Adobe Audition (Adobe Inc., San Jose, CA). They averaged 593 ms in length.

Each trial began with a centrally presented fixation cross for a time-interval jittered between 250 and 500 ms. While the cross stayed on for another 1000 ms, a spoken word was presented binaurally at approximately 65 dB SPL via non-magnetic earpieces (Etymotics ER2 Acoustic Stimulator) driven through 2 m plastic tubes^1^. The spoken word was followed by a blank screen of 1500 ms. On the majority of trials the participant made no response and was asked to simply listen attentively to the spoken words. On 8% of the trials, chosen at random, a written probe word was presented after the blank screen. On these trials (designed to ensure the participants were paying attention) the participants performed a one-back memory task, indicating whether the visually presented word matched the preceding acoustic stimulus or not by pressing a response button. Half of the participants answered “yes” with the right hand and “no” with the left hand. The other half used the reverse combination. Feedback was presented on the screen for 1000 ms and followed by a blank screen of 500 ms. The presentation and timing of stimuli was controlled using Eprime software (www.pstnet.com). Each item was presented twice in a pseudorandom order within 7 blocks. Each participant received 20 practice trials, which included a presentation of each different stimulus type and three exemplars of one-back memory trials.

### Data recording

Continuous MEG data were recorded using a 306 channels VectorView system (Elektra-Neuromag, Helsinki, Finland) containing 102 identical sensor triplets, composed of two orthogonal planar gradiometers and one magnetometer, covering the entire head of the subject. Participants sat in a dimly lit magnetically-shielded room (IMEDCO AG, Switzerland). The position of the head relative to the sensor array was monitored continuously by feeding sinusoidal currents into four Head-Position Indicator (HPI) coils attached to the scalp. EEG was recorded simultaneously from 70 Ag-AgCl electrodes placed within an elastic cap (EASYCAP GmbH, Herrsching-Breitbrunn, Germany) according to the extended 10/20 system and using a nose electrode as the recording reference. Vertical and horizontal EOG were also recorded. All data were sampled at 1 kHz with a band-pass filter from 0.03 to 330 Hz. A 3D digitizer (Fastrak Polhemus Inc., Colchester, VA) was used to record the locations of the EEG electrodes, the HPI coils and approximately 50–100 “headpoints” on the scalp, relative to three anatomical fiducials.

### Data pre-processing

Static MEG bad channels were detected and excluded from all subsequent analyses using MaxFilter (Elektra-Neuromag). Compensation for head movements (measured by HPI coils every 200 ms) and a temporal extension of the signal–space separation technique (SSS) was applied to the MEG data using MaxFilter. Static EEG bad channels were visually detected and interpolated (Hämäläinen and Ilmoniemi, 1994). The EEG data were re-referenced to the average over all channels. The continuous data were low-pass filtered to 40 Hz and epoched with respect to the onset of each word containing the first 200 ms period of the stimuli. By analysing the neural response to the earlier part of word, we hope to minimize the potential influence of high-level linguistic and cognitive processes (Hauk et al., 2012). Baseline correction was applied by subtracting the average response of the 100 ms prior to onset of the epoch. EEG and MEG epochs in which the EEG or EOG exceeded 200 μV, or value on any gradiometer channel exceeded 2000 fT/m were rejected as potentially containing artifacts. In addition, artifact components associated with eye-blinks and saccades were automatically detected and removed using the independent component analysis tools of EEGLAB (Delorme and Makeig, 2004).

### Source reconstruction

We estimate the location of cortical sources with the anatomically constrained minimum norm estimate (MNE; Hämäläinen and Ilmoniemi, 1994). MR structural images were obtained using a GRAPPA 3D MPRAGE sequence (*TR* = 2250 ms; *TE* = 2.99 ms; flip-angle = 9°; acceleration factor = 2) on a 3 T Trio (Siemens, Erlangen, Germany) with 1 mm isotropic voxels. From the MRI data, a representation of each participant's cerebral cortex was constructed using the FreeSurfer program (http://surfer.nmr.mgh.harvard.edu/). The forward model was calculated with a three-layer Boundary Element Model (BEM) using the outer surface of the scalp as well as the outer and inner surfaces of the skull identified in the anatomical MRI. This combination of MRI, MEG, and EEG data provides better source localization than MEG or EEG alone. The constructed cortical surface was decimated to yield approximately 12,000 vertices that were used as the locations of the dipoles. To perform group analysis, the cortical surfaces of individual subjects were inflated and aligned using a spherical morphing technique implemented by MNE (Gramfort et al., 2014). Sensitivity to neural sources was improved by calculating a noise covariance matrix based on the 100 ms pre-stimulus period. The activations at each location of the cortical surface were estimated over 1 ms windows.

## Spatiotemporal searchlight representational similarity analysis (ssRSA)

In ssRSA, we need to represent the similarity structure in the observed dynamic patterns of brain activation as well as the theoretically relevant similarity structure in the stimuli (Su et al., 2012). As noted earlier, the former is called the data RDM and the latter is called the model RDM. Brain data RDMs express the pairwise similarity between neural activation patterns in the EMEG data. In general, a model RDM expresses a hypothesis about what the neural system might encode. If the brain data RDM matches the model RDM, we can infer that the cortical region from which the brain data RDM is derived may indeed code information captured by the model RDM. If there is only one hypothesis about the data, the simplest approach to determine the match between data and model RDMs is to compute a Spearman's correlation between them (e.g., Su et al., 2012). This approach is not used in the current study, since there are multiple interrelated hypotheses—i.e., several model RDMs each of which represents a hypothesis about a particular frequency band in the sound. This is because (as specified below) we generate a model RDM for each of sixteen frequency components in the stimuli ranging from 30 to 8000 Hz. Instead of Spearman's correlation, we used a general linear model (GLM) approach to estimate the contribution of each hypothesis (as expressed in each model RDM) in explaining the brain data RDM. The GLM estimates a set of parameters, which show how well each model RDM matches the data. The advantage of this approach is that the comparisons between brain data RDM and multiple model RDMs are performed in a single step, and GLM can also consider correlations between different model RDMs while estimating the relevant parameters (Mur et al., 2013).

The ssRSA procedure extends the concept of spatial searchlight developed for fMRI to include an extra temporal dimension (Su et al., 2012). This is done by combining a spatial searchlight with a sliding time window under the assumption that neuro-cognitive representations may be realized in the continuous spatiotemporal patterns of the source-reconstructed EMEG data. Specifically, at each spatial location (or vertex) on the MR-estimated cortical surface, the searchlight covers a hexagonal cortical patch (approximately 20 mm radius), which includes about 128 vertices. Based on previous research (e.g., Moerel et al., 2012), we predefined a search area that covered bilateral superior temporal cortex, including Heschl's gyrus, superior temporal gyrus (STG), and superior temporal sulcus (STS) using Freesurfer cortical parcellation (Fischl et al., 2004; Desikan et al., 2006) (see **Figure 7** below).

In this study, we analyzed the data from the onset of the word to 200 ms after onset. The choice of this short time period reduces the potential influence of linguistic processing, providing a clearer view of auditory cortical organization. The temporal dimension of the ssSRA searchlight was set to 30 ms in width and was moved, as a sliding window, from word onset to 200 ms after onset in incremental steps of 10 ms. These parameters were chosen to give sufficiently fine-grained temporal resolution to reveal the development of neural responses in the brain. The same sliding time window was applied to the EMEG data, when computing the brain data RDM, as was applied to the stimuli when constructing the model RDMs. This allowed us, when comparing a model RDM to a data RDM, to compare RDMs derived from the same time period.

The brain data RDM was computed for each spatiotemporal searchlight location and assigned to the center vertex of the hexagonal searchlight region. We allow the searchlight to overlap in space and in time resulting in separate brain activation RDMs for each vertex at each time point. Sampling with overlapping spatiotemporal searchlights enables us to detect distributed and transient representations that might otherwise straddle the boundary between adjacent cortical patches or successive temporal windows and fail to be analyzed as a single pattern.

The data RDM for each vertex and each time point was computed for each subject individually, and an averaged RDM was then derived across all subjects. This averaging at the level of dissimilarity rather than at the level of neural response allows ssRSA to take into account individual variability in neural representations. This method is less affected by differences in how a stimulus is actually encoded in a subject's auditory cortex, because while the same auditory stimulus may elicit a unique EMEG pattern for that specific individual (Moerel et al., 2013), we can nonetheless assume that the similarity structure across the 400 words is directly comparable across subjects.

We explain below how we compute brain data RDMs based on the EMEG data and model RDMs based on human cochlear models of the stimuli. We then describe how we estimate parameters from the GLM relating brain data and model RDMs, and how we map frequency preference and selectivity across human auditory cortex using ssRSA.

### Constructing brain data RDMs

**Figure 1:**
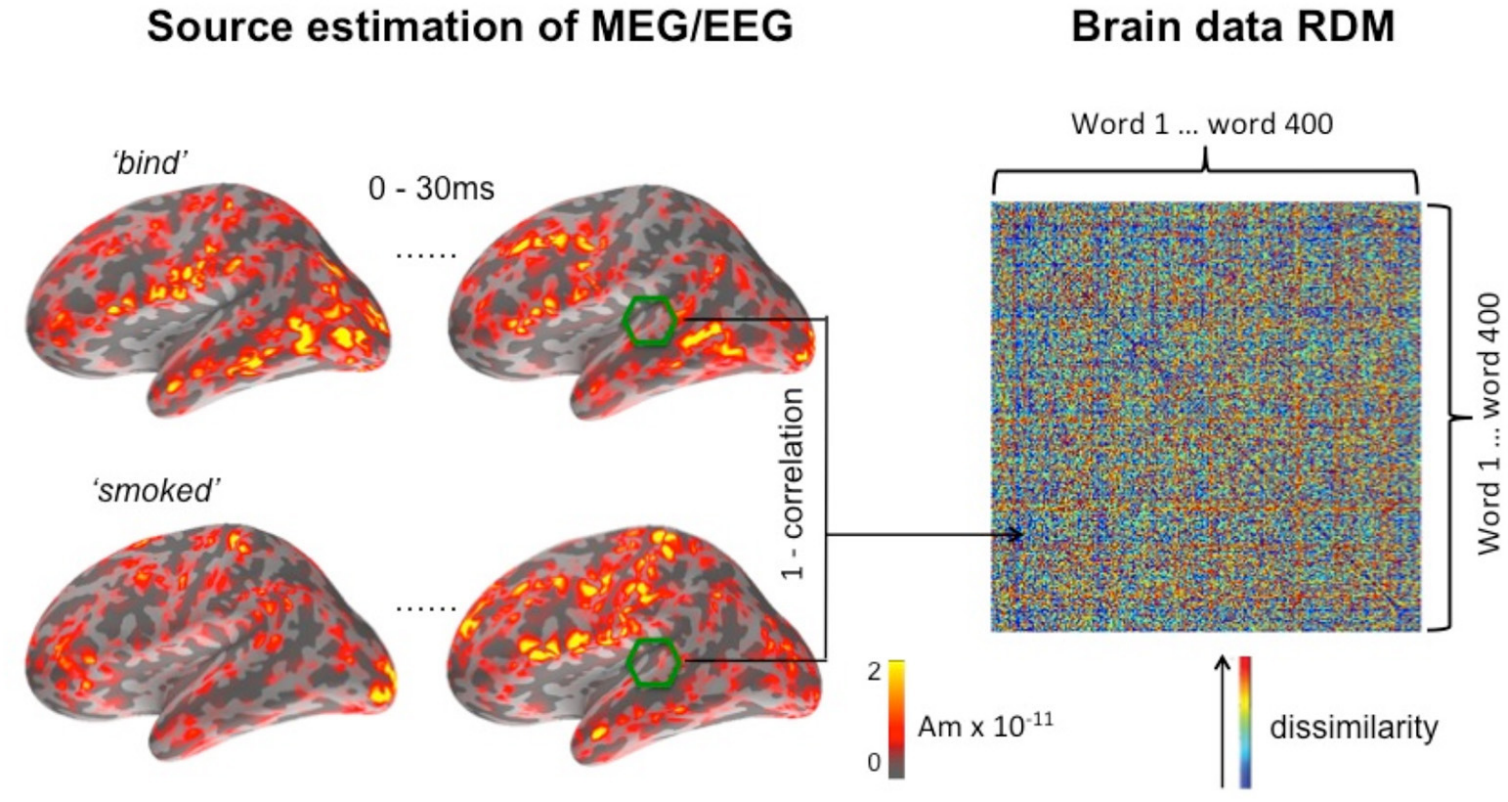
**Computing the brain data RDM for a searchlight window centered on a vertex in or near auditory cortex in the left hemisphere**. The temporal dimension of the window is set at 30 ms, running here from the onset of the word, and its spatial dimension is set at a radius of 20 mm. EMEG source activation for this window is shown for two of the 400 words (“bind” and “smoked”) making up the full matrix.

As previously discussed, a brain data RDM is derived from the dynamic patterns of neural activation over space and time. Each entry in the RDM is a correlation distance (1 minus the correlation value) between the activation patterns elicited by a pair of experimental conditions (here, individual words). These activation patterns are the source estimations of the EMEG data for each pair of words, as computed within a window defined by the searchlight algorithm (see Figure 1). In general, such a pattern is based on the distribution of EMEG source estimation over a number of vertices over a period of time. For the same group of vertices and the same time window, the pattern of activation will differ between conditions (pairs of words) because the underlying neural population responds differently to different auditory stimuli. If two stimuli are similar in their physical properties, e.g., sounds with similar frequency components, then the distribution of source estimations for these stimuli should be more similar in auditorily sensitive cortex. Conversely, two distinct auditory stimuli should elicit more dissimilar activation patterns in these regions.

As previously implemented in the fMRI variant of RSA (Kriegeskorte et al., 2006), we define similarity between two activation patterns as the Pearson's correlation distance between the two data vectors of EMEG source estimations. Here, the data vector refers to a one-dimensional vector derived from the activation pattern, which is a three-dimensional object (two-dimensions for space along the cortical surface and a dimension of time). Figure 1 shows an example brain data RDM computed from a location in the superior temporal lobe. The center of the searchlight was placed in the region of auditory cortex, with a radius of 20 mm, and a time window that runs 30 ms from the onset of the stimulus. The example shown captures the initial response in the auditory cortex, since the latency of a detectable signal in the auditory core area is approximately 10 ms post-stimulus onset in animals (e.g., Heil, 1997). By using a sliding time window approach for the generation of model and brain data RDMs, we can track the unfolding of this response over time, while the searchlight method covers the entire search area by moving the center of the searchlight vertex-by-vertex throughout this area.

On the right hand side of Figure 1, it can be seen that elements on the main diagonal of RDM are zeros by definition. In the off-diagonal parts of the matrix, a large value (shown in red) indicates that the two conditions have elicited highly dissimilar spatiotemporal activation patterns, and vice versa for small values (shown in blue). The RDM is a 400 × 400 matrix representing the pairwise similarity between the 400 words used in the experiment (ordered alphabetically). RDMs computed using this method are symmetric about the main diagonal, and subsequent computations were restricted to the portion of the matrix falling above the diagonal.

### Theoretical model RDMs

The motivation for generating model RDMs to map tonotopic distributions is in order to make predictions about the spatiotemporal patterns induced in EMEG data by the frequency dimension of a stimulus set. The performance of the ssRSA procedure is specific to the hypotheses being tested and will only show effects that match the specified model RDMs. To keep the model RDMs as realistic as possible, as a reflection of the spectral characteristics of early auditory processes, the stimuli were filtered using a Gammatone filter bank (Patterson, 1987) to generate a probable representation of the spectral response for each word, as output by the human cochlea. This representation was chosen because of the inherent bias of the human ear to different frequency components in the auditory stimuli, and because the Gammatone filter bank model has been validated by numerous empirical investigations (e.g., Patterson, 1987).

**Figure 2:**
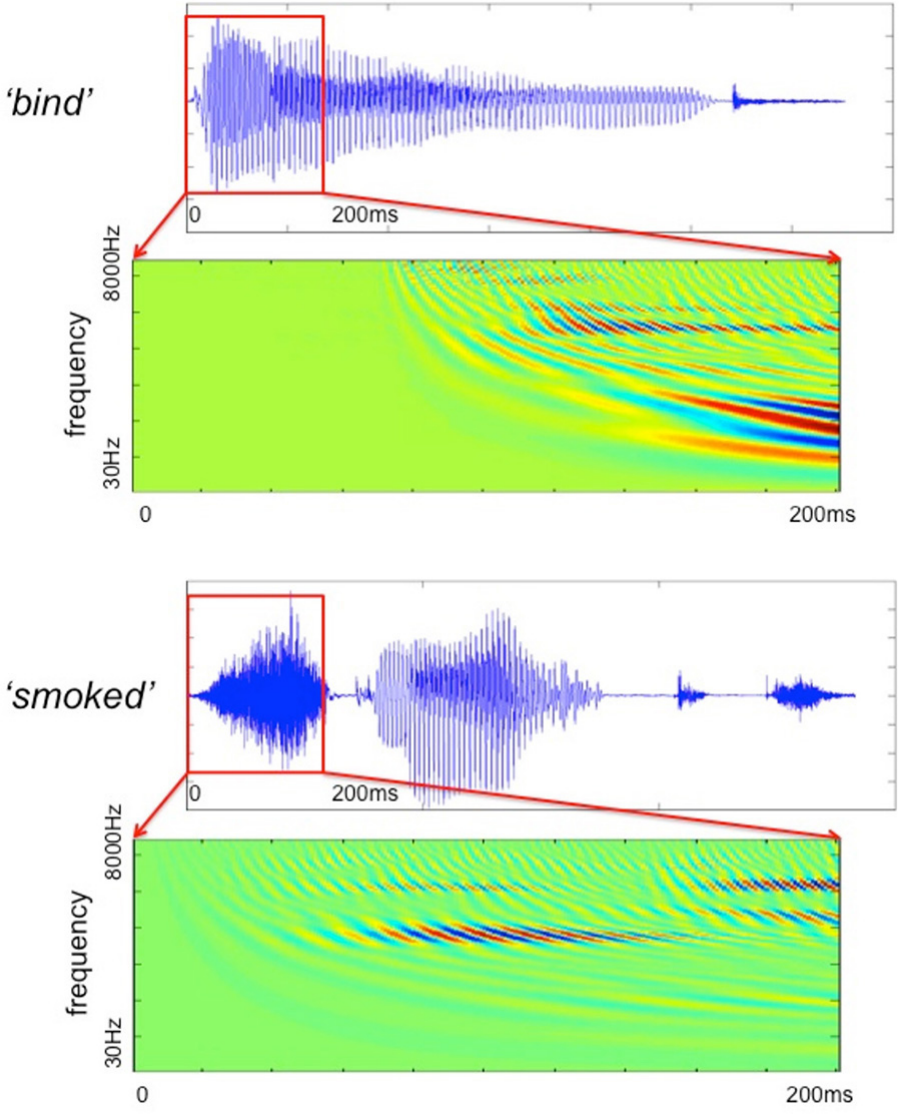
**The Gammatone filter models computed for two stimuli (bind and smoked) from word onset to 200 ms after onset**.

The filter bank aims to generate a cochlear representation of an auditory signal by convolving the signal with the impulse responses of individual Gammatone filters. The envelope of each filter is designed in such a way that it is narrow at low frequency channels and the impulse responses are wider for high frequencies (Patterson, 1987). We used an implementation of 4th order non-phase aligned Gammatone filters in Matlab by Christopher Hummersone (http://www.mathworks.co.uk/matlabcentral/fileexchange/32212-gammatone-filterbank). The center frequency of each channel is equally spaced between low and high center frequencies on the Equivalent Rectangular Bandwidth (ERB) scale. As previously mentioned, we set the lowest frequency channel to 30 Hz and the highest to 8000 Hz. These values were chosen based on the power spectral density of the stimuli such that most of the energy of the sound was covered by our analysis. The total number of frequency channels was set to 128 in order to cover the frequency range from 30 to 8000 Hz with sufficient resolution without losing spectral information. This resulted in a cochleagram that represents activation across time for each of the 128 frequency channels. Table 1 gives the center frequencies of these frequency channels, and Figure 2 provides two examples of cochleagrams computed from word onset to 200 ms post-onset. The model RDMs are computed by sampling from these cochleagram in a 30 ms time window moved in incremental 10 ms steps over this 200 ms epoch.

**Table 1:** **Center frequencies for 128 Gammatone filters grouped into 16 bands ranging from 30 to 8000 Hz (equally spaced on a logarithmic scale)**.

	**Center frequencies of Gammatone filters (Hz)**							
Band 1	30.0	37.1	44.5	52.0	59.8	67.8	76.0	84.4
Band 2	93.0	101.9	111.0	120.4	130.1	140.0	150.2	160.6
Band 3	171.4	182.4	193.8	205.5	217.4	229.8	242.4	255.4
Band 4	268.8	282.6	296.7	311.2	326.1	341.4	357.2	373.3
Band 5	390.0	407.1	424.6	442.7	461.2	480.3	499.8	520.0
Band 6	540.6	561.9	583.7	606.2	629.2	652.9	677.2	702.3
Band 7	728.0	754.4	781.5	809.4	838.1	867.6	897.8	929.0
Band 8	960.9	993.8	1027.5	1062.2	1097.9	1134.5	1172.2	1210.8
Band 9	1250.6	1291.4	1333.4	1376.6	1420.9	1466.4	1513.3	1561.4
Band 10	1610.8	1661.6	1713.8	1767.4	1822.5	1879.2	1937.4	1997.2
Band 11	2058.7	2121.8	2186.7	2253.4	2322.0	2392.4	2464.8	2539.2
Band 12	2615.6	2694.1	2774.9	2857.8	2943.0	3030.6	3120.6	3213.1
Band 13	3308.1	3405.8	3506.2	3609.3	3715.3	3824.2	3936.1	4051.1
Band 14	4169.3	4290.7	4415.5	4543.7	4675.5	4810.9	4950.1	5093.1
Band 15	5240.1	5391.1	5546.2	5705.7	5869.6	6038.0	6211.0	6388.8
Band 16	6571.5	6759.3	6952.3	7150.6	7354.3	7563.7	7778.9	8000.0

**Figure 3:**
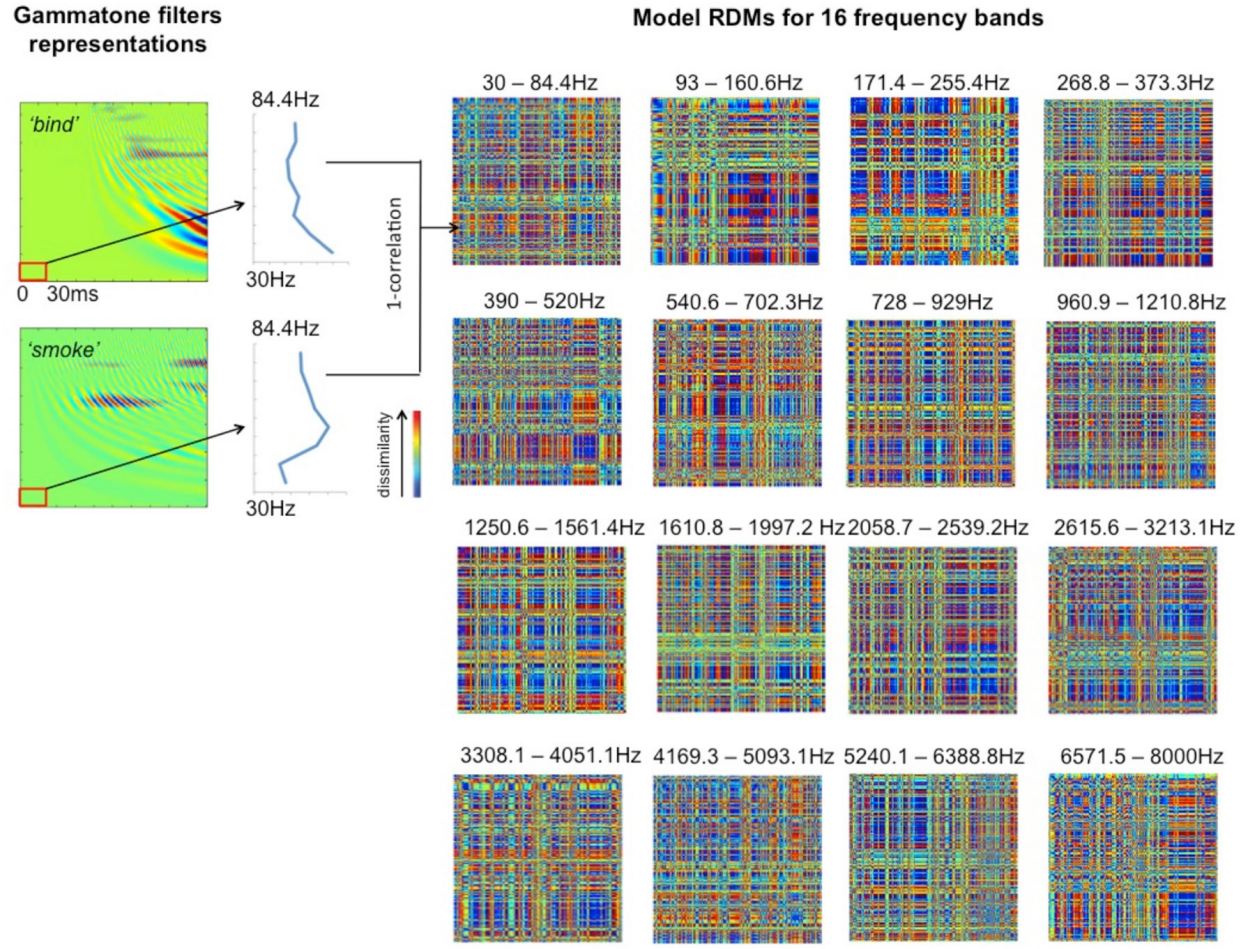
**Model RDMs for 16 frequency bands (right hand panels), based on the first 30 ms from stimulus onset, and representing the pairwise correlations between the 400 stimulus words in a 400 × 400 matrix**. The **left hand panels** show two illustrative cochleagrams (see Figure 2), sampled at the lowest frequency band over the given time window. The vertical blue lines indicate the frequency characteristic curve for each word for the band indicated.

To explore how auditory cortex responds to different frequency components, we divided each cochleagram into 16 frequency bands with 8 channels in each band, as shown in Table 1. This allows us to preserve the rich frequency-varying properties of the stimuli while reducing the number of model RDMs that need to be tested, thereby improving the efficiency of the ssRSA algorithm. The selected 30 ms time window moves with an incremental step of 10 ms in order to capture the detailed dynamical changes of spectral information in the stimuli. Within each frequency band, at each incremental time step, we generate a frequency-characteristic curve by taking the mean energy of all constituent frequency channels across time and converting this into magnitude gain in dB. A model RDM is built up by computing, for each pair of frequency bands and time windows, the pairwise correlation distance (1—Pearson's correlation) using the corresponding frequency characteristic curves for the two words in question (see Figure 3, left panel). In this way, each full model RDM reflects the similarity between all 400 stimuli at a particular combination of frequency bands and time points. Our key hypothesis here is that if a brain data RDM matches one of these model RDMs, then the brain region from which the data RDM is derived may encode information for the corresponding frequency band. Figure 3 shows 16 model RDMs for different frequency bands calculated for a time window of 0–30 ms from the onset of the stimuli.

### Comparing brain data RDMs to theoretical model RDMs

**Figure 4:**
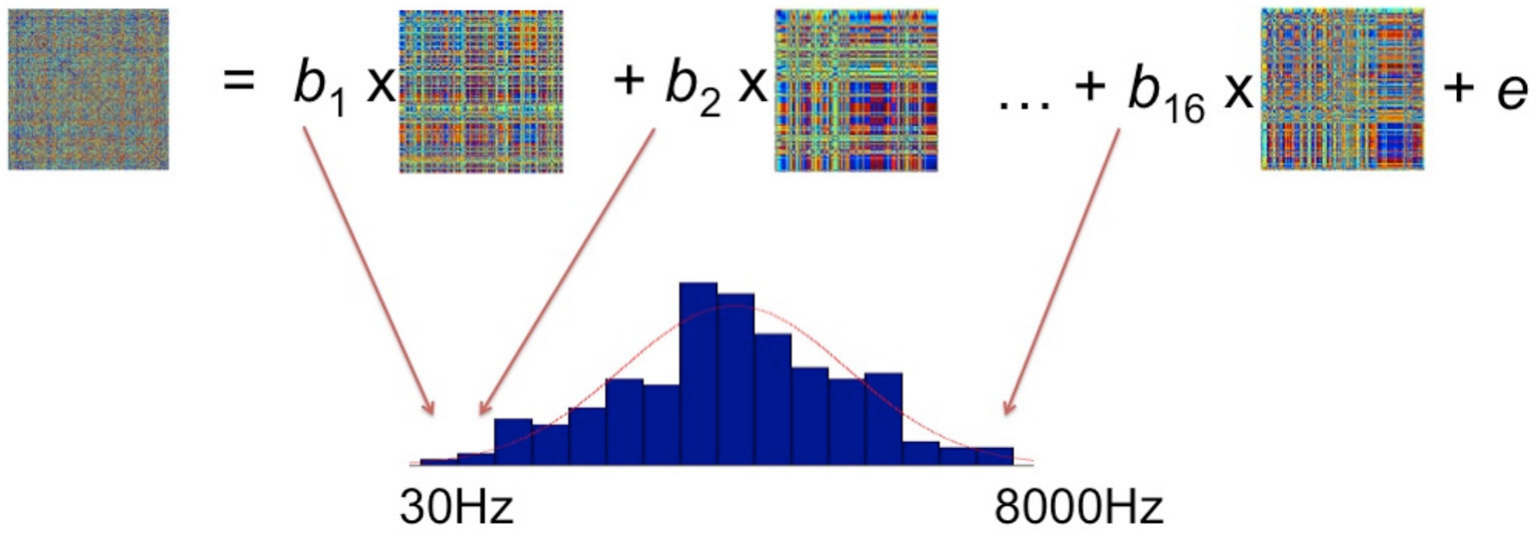
**Fitting the brain activation RDM with 16 model RDMs (each of which corresponds to a particular frequency range) using the General Linear Model**. The resulting beta parameters were used to determine a Gaussian tuning curve (red curve).

From the sequence of operations outlined in the preceding sections, we obtain a set of model RDMs, which define a set of hypotheses about potential stimulus properties, and a set of brain data RDMs averaged over subjects for every vertex and time point as the searchlight moves. The comparisons between brain activation RDMs and model RDMs were performed by fitting a GLM as shown in Figure 4. In the GLM, the data RDM is expressed by a linear combination of 16 model RDMs, each representing a different frequency band. Estimating the GLM gave 16 beta parameters and a residual matrix for this particular vertex for a particular time window. These parameters reflect how much variance in the brain activation RDM can be explained by the corresponding frequency component in the stimuli. Since neurons in the brain do not respond only to a single frequency but potentially to a range of different frequencies (e.g., Moerel et al., 2012, 2013), we fit a Gaussian distribution to the 16 estimated parameters. We suggest that this Gaussian is akin to the tuning curve found in neurophysiology representing the frequency preference and selectivity of the underlying neural population. After we have fitted the Gaussian distribution, we assign the center and the standard deviation (*SD*) of this Gaussian to the centroid of this searchlight (Formisano et al., 2003; Moerel et al., 2012). In the presentation of the results below (**Figures 6–10**) we focus on these measures of frequency response in order to preserve comparability with the Moerel et al. (2012) results. In addition, to give a more complete picture of the sensitivity of the EMEG/ssRSA technique, we also provide information about the distribution of the *peak* frequencies observed for each Gaussian (see **Figure 8**).

Note that this computation of the Gaussian tuning curve for any specific vertex at a single time window (e.g., 40–70 ms), as shown in Figure 4, will take into account the match between this data RDM and the model RDM that has the same start and end points as this time window. The result of the GLM is assigned to the mid-point (i.e., 55 ms) of the sliding time window under analysis. In the next overlapping position of the sliding time window (i.e., 50–80 ms), we carry through the same procedure for a new pair of data and model RDMs computed for this new time window. The result of this GLM is assigned to the 65 ms mid- point for this window.

More generally, it is important to note that we assume—in common with the field in general—that the functional properties of auditory cortex are relatively stable over time, and certainly over the 200 ms epoch employed here. This means that the tonotopic maps generated at each time window are providing information about the properties of the same underlying object of interest—in this study the organization of frequency-sensitive processes in bilateral human superior temporal cortex. At the different time windows sampled here, however, the varying frequency properties of the stimuli will be different, so that the model RDMs probe the auditory system with differing spectral context across time windows. In this sense, every time-point can be seen as a new test for auditory cortex.

At the endpoint of the ssRSA process, which has generated Gaussian frequency tuning curves at every vertex in the superior temporal cortex search area at every incremental time point from onset to 200 ms, it is necessary to combine this information to give a unified view of the functional organization of these brain areas. We do this by averaging the center frequencies and the SDs across the 200 ms analysis epoch at each vertex. These values are then mapped back to the brain, so that the results of the ssRSA analysis are brain maps showing the frequency preference reflected by the center of the Gaussian and the frequency selectivity reflected by the SD. It is these averaged data, appropriately statistically thresholded, that we present in the Results Section below ^2^.

### Correction for multiple comparisons

In the source estimations of EMEG data, there can be tens of thousands of vertices multiplied by hundreds of time points. The analysis will therefore contain very large numbers of individual comparisons even within our relatively restricted search area (bilateral superior temporal cortex). This creates a massive spatiotemporal multiple comparisons problem, potentially resulting in high proportions of false positives. To assess the significance of the results and to control for false positives, we therefore performed permutation-based statistical tests (Bullmore et al., 1996; Brammer et al., 1997; Nichols and Holmes, 2001). Under the assumption of the null hypothesis, the superior temporal cortex does not represent the frequency properties of a sound, so that any two sounds are equally similar in terms of their neural response to frequency. If the null hypothesis holds, we can freely relabel each condition, and the relationship between our model RDMs and the condition-relabeled brain activation RDMs would not change.

To simulate the null hypothesis, we randomly permuted the condition labels of our 400 stimuli by swapping the rows and columns of all brain data RDMs. When performing this random permutation, we kept the order of the permutation unchanged for all data RDMs across vertices and time points in order to preserve the spatiotemporal autocorrelation in the data. We performed 1000 permutations of our data RDMs. For each permutation, we compared the new data RDMs with the same set of model RDMs using GLM as we did with the original data. We then selected the maximum beta parameters for each GLM. From these 1000 permutations, we were able to build a null distribution of the maximum beta parameters (28,028,000 data points in total), which assumes that the fit between data and model RDMs was due only to random noise. Thresholding the null distribution to select the top 5% will ensure that we have a risk equivalent to *p* = 0.05 of detecting any vertex as significant (i.e., as a false positive) if the null hypothesis were true. We therefore control false positives by thresholding our tonotopic maps with the appropriate beta value that picks out the top 5% of the null distribution. This procedure addresses the spatiotemporal multiple comparisons problem, and ensures that the results are robust to noise.

## Results

### The null distribution

**Figure 5:**
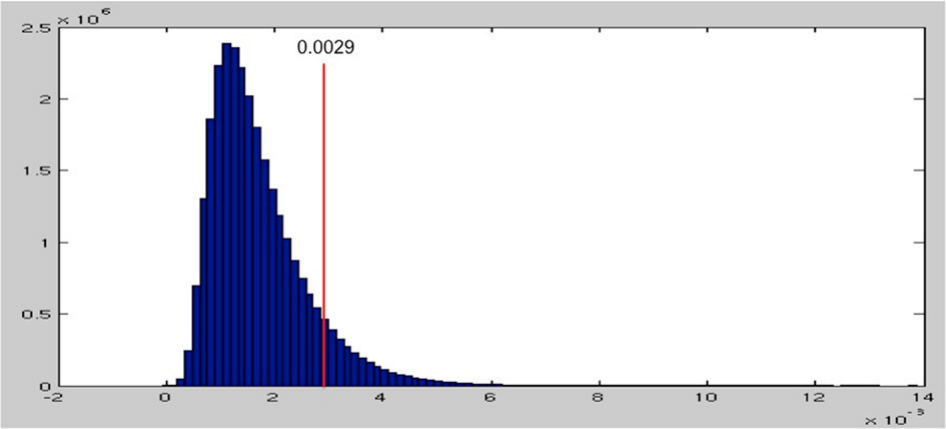
**The null distribution of maximum beta parameters from 1000 permutations**. The threshold for the top 5% is at 0.0029, shown as the red line.

After permuting the condition labels in our data RDMs 1000 times, we obtain a null distribution of the maximal beta parameters (see Figure 5). This null distribution is skewed toward zero and has a long tail toward the positive end. This distribution reflects the fact that when we randomly permute the condition labels, thereby disassociating the EMEG data of each word from their spectral characteristics, most of the model RDMs fail to explain much of the variance in the permuted data RDMs, resulting in beta values close to zero. This in fact is what the null hypothesis is assuming. Thus, values above zero reflect false positives and *p* = 0.05 corresponds to the threshold of beta values at 0.0029, which selects the top 5% of the null distribution.

### Frequency response distributions

**Figure 6:**
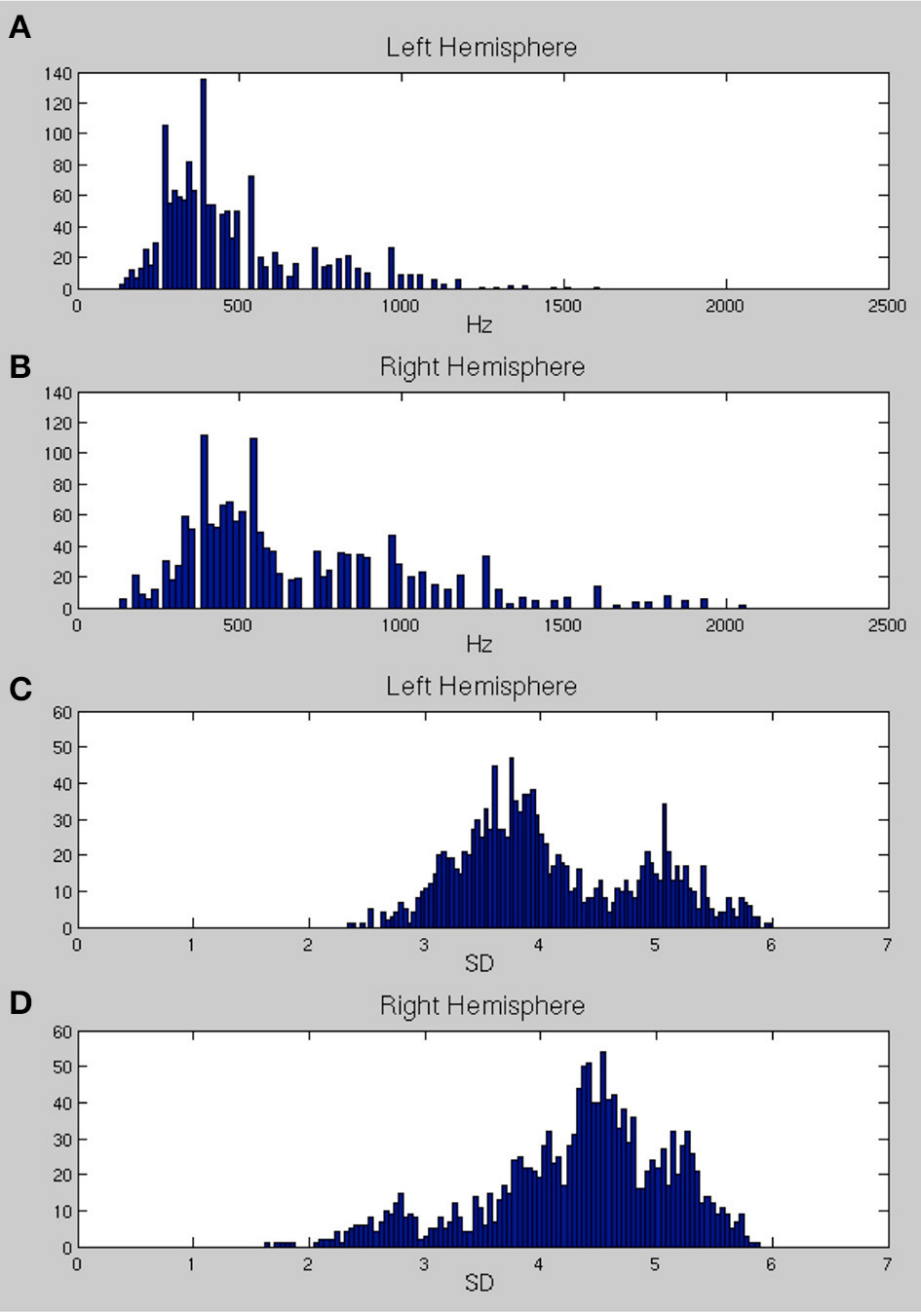
**(A,B)** The distribution of center frequencies (in Hz) for all vertices in bilateral superior temporal cortex (**A**, left hemisphere and **B**, right hemisphere). **(C,D)** The distributions of the standard deviation (*SD*) of the Gaussian tuning curve, expressed in frequency band units, for all vertices in bilateral temporal cortex (**C**, left; **D**, right).

Figures 6A,B show the distributions of preferred frequencies for all vertices in bilateral superior temporal cortex, summarizing the results compiled over the 200 ms from stimulus onset. These distributions are very similar for the two hemispheres although the right hemisphere shows a small shift toward higher frequencies. The peak of the distribution on the left is at around 250–350 Hz, which is in the range of the fundamental frequency of the female voice (the stimuli were recorded by a female speaker). The peak for the right hemisphere was slightly higher, at 450 Hz. The distribution of frequency preferences for both hemispheres was bimodal with a second (much reduced) peak centered at around 900–1000 Hz, likely reflecting the harmonic formant structures in speech. This result suggests that the majority of the area sampled (extending well beyond primary auditory cortex as standardly defined) responded most strongly to the predominant frequencies in the voice range (200–2000 Hz). Note that this did not mean an absence of responses to higher frequencies, as can be seen by the peak frequency plots provided in **Figure 8**.

**Figure 7:**
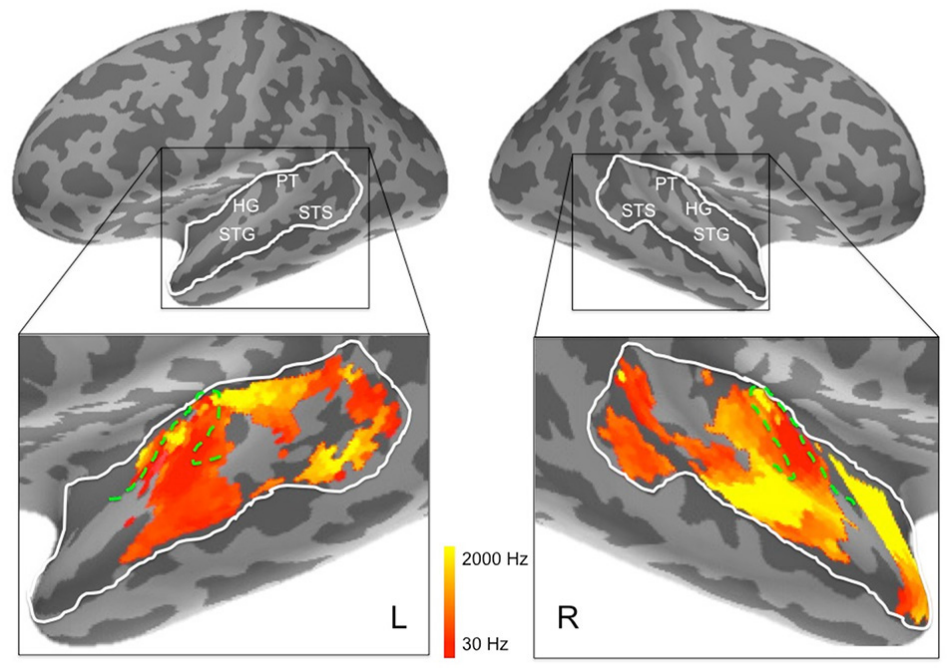
**Frequency preferences in left and right hemisphere superior temporal cortex derived from ssRSA analysis of EMEG data**. The search areas were restricted to the regions denoted by the white lines in the upper panels. Green dashed lines (lower panels) show the outlines of Heschl's gyrus (HG). Other anatomical landmarks are superior temporal gyrus (STG), superior temporal sulcus (STS), and planum temporale (PT). The outlines of HG were generated based on the FreeSurfer cortical parcellation (Fischl et al., 2004; Desikan et al., 2006) and on Moerel et al. (2014).

Figures 6C,D show the distributions of the standard deviation (*SD*) of the Gaussian tuning curve for each vertex in bilateral superior temporal cortex. Note that because the unit of the SD is expressed in frequency bands, the SDs for Gaussians with higher frequency preferences will cover larger ranges of frequencies (in Hz). The distributions for the left hemisphere are strongly bimodal, with a narrow selectivity group centered around a peak SD distribution of 3.7, and a broader sensitivity group with a peak SD distribution of 5.2. The right hemisphere (Figure 6D) shows a very different pattern, with most of the region tested being only weakly frequency selective. There is a primarily unimodal distribution, corresponding to the broader sensitivity group in the left hemisphere, with the peak of the SD distribution falling at 4.5. A much smaller set of vertices show stronger selectivity, with SDs falling in the range 2–3. The spatial mapping of frequency preference and selectivity is shown in Figures 7, **9** below.

### Maps of frequency preference (center and peak frequencies)

The results of the ssRSA procedure, after applying the threshold derived from the permutation testing, were two maps of bilateral human superior temporal cortex. The first map shows frequency preference (the center frequency of the Gaussian tuning curve) and the second shows frequency selectivity (the SD of the tuning curve). In both maps we see frequency sensitive processes extending across the entire area of interest, and well outside primary auditory areas (Heschl's gyrus, planum temporale, etc.). Figure 7 shows that in the left hemisphere, regions exhibiting higher center frequency preferences (yellow) are seen medially in Heschl's gyrus, with a substantial area of higher frequency preference extending posteriorly into planum temporale and posterior STG. Further higher frequency sensitive areas, at some distance from primary auditory cortex, are found more ventrally in posterior STS. Low frequency regions (darker orange/red) occur in an extensive region of the middle part of the superior temporal lobe, extending laterally and ventrally from Heschl's gyrus into STG and STS. Heschl's gyrus itself appears to exhibit the high-low and low-high tonotopic gradients frequently reported for this region in earlier studies (Baumann et al., 2013; Saenz and Langers, 2014)—see below for further discussion.

In the right hemisphere, Heschl's gyrus exhibits primarily lower center frequency preferences—similarly to the left hemisphere—with an apparent low-high tonotopic gradient extending postero-medially. A pronounced higher center frequency preference area is located lateral to Heschl's gyrus, extending to STS. A further higher center frequency preference strip is seen in anterior STG and STS, reaching as far as the temporal pole. No comparable antero-medial region of frequency sensitivity is seen on the left. The right hemisphere also shows a substantial region more posteriorly of lower center frequency preferences, falling mainly in posterior STS. It should be noted, however, that center frequency preferences in right superior temporal cortex are generally accompanied by broadly tuned frequency selectivity (see **Figure 9**).

**Figure 8:**
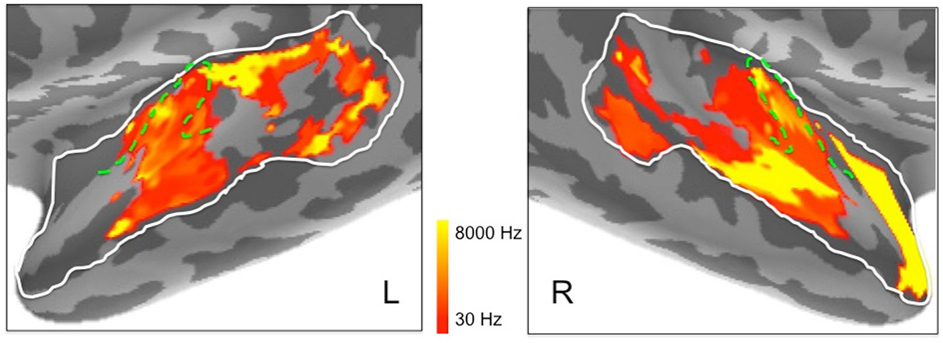
**Peak frequencies in left and right hemisphere superior temporal cortex derived from ssRSA analysis of EMEG data plotted in logarithmic scale in order to accommodate the full frequency range**. Dashed green lines show the outlines of Heschl's gyrus (HG).

In addition, to complement these plots of center frequency preference for each vertex, we also provide plots of the significant peak frequencies observed for the same vertices (see Figure 8). These plots, showing patches of peak frequency extending up to 8000 Hz, show a very similar distribution across superior temporal cortex to the center frequency results in Figure 7. Unsurprisingly, when the Gaussian tuning curve includes responses to lower frequencies, this may shift the center frequency of the Gaussian (see Figure 7) downwards from the peak frequency (Figure 8).

### Maps of frequency selectivity

**Figure 9:**
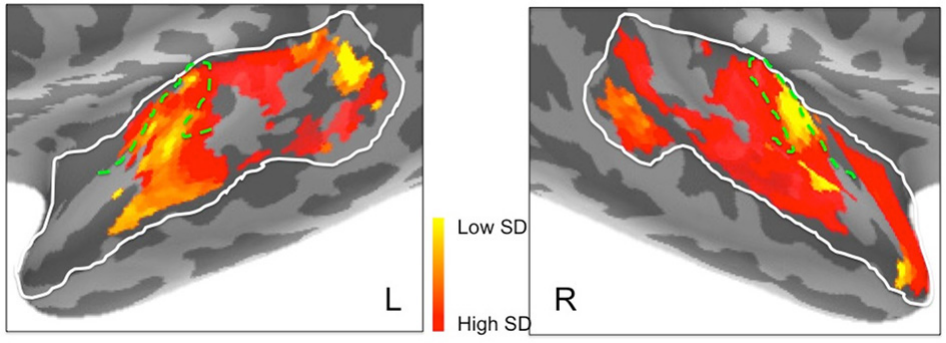
**Frequency selectivity in left and right hemisphere superior temporal cortex**. Areas in yellow, with low standard deviation (*SD*), represent brain regions that are selective to a narrower band of frequencies. Dashed green lines show the outlines of Heschl's gyrus.

Figures 6C,D suggested marked differences between the hemispheres in frequency selectivity. This is reflected in the spatial maps of frequency selectivity (Figure 9). The right hemisphere shows relatively broad frequency selectivity (dark orange/red) across almost all of the regions tested. Only Heschl's gyrus, indicating primary auditory cortex, shows a substantial patch of narrow frequency selectivity (yellow), corresponding to the region of low frequency preference seen in Figure 7. The left hemisphere shows generally narrower frequency selectivity (light orange to yellow), with an area of narrow selectivity also falling into Heschl's gyrus, in a comparable location to the right. There is also a marked and well-defined area of narrow selectivity in posterior STG and STS, which is not present on the right.

## Discussion

### Organization of auditory cortex

The ssRSA analyses of real-time neural responses to spectrally complex spoken words, as summarized in Figures 6–9, seem to deliver statistically robust and regionally coherent patterns of frequency sensitivity and frequency selectivity across bilateral superior temporal cortex. The critical issue in evaluating these outcomes, however, is their interpretability relative to existing data and theory where human auditory cortex is concerned, as well as to the analyses of tonotopy carried out in non-human primates using invasive methods.

We present below some initial comparisons along these lines, but we emphasize that these comparisons can only be preliminary. A more quantitative treatment of the parallels between the current ssRSA/EMEG results and the high-field fMRI results will require a deeper analysis of how the very different properties of the two analysis processes might affect how variations in frequency preference and selectivity are captured by each process, and the consequences of this for spatial maps of these variations. This analysis is outside the scope of the current paper, and the comparisons we provide below should be regarded as preliminary and illustrative.

**Figure 10:**
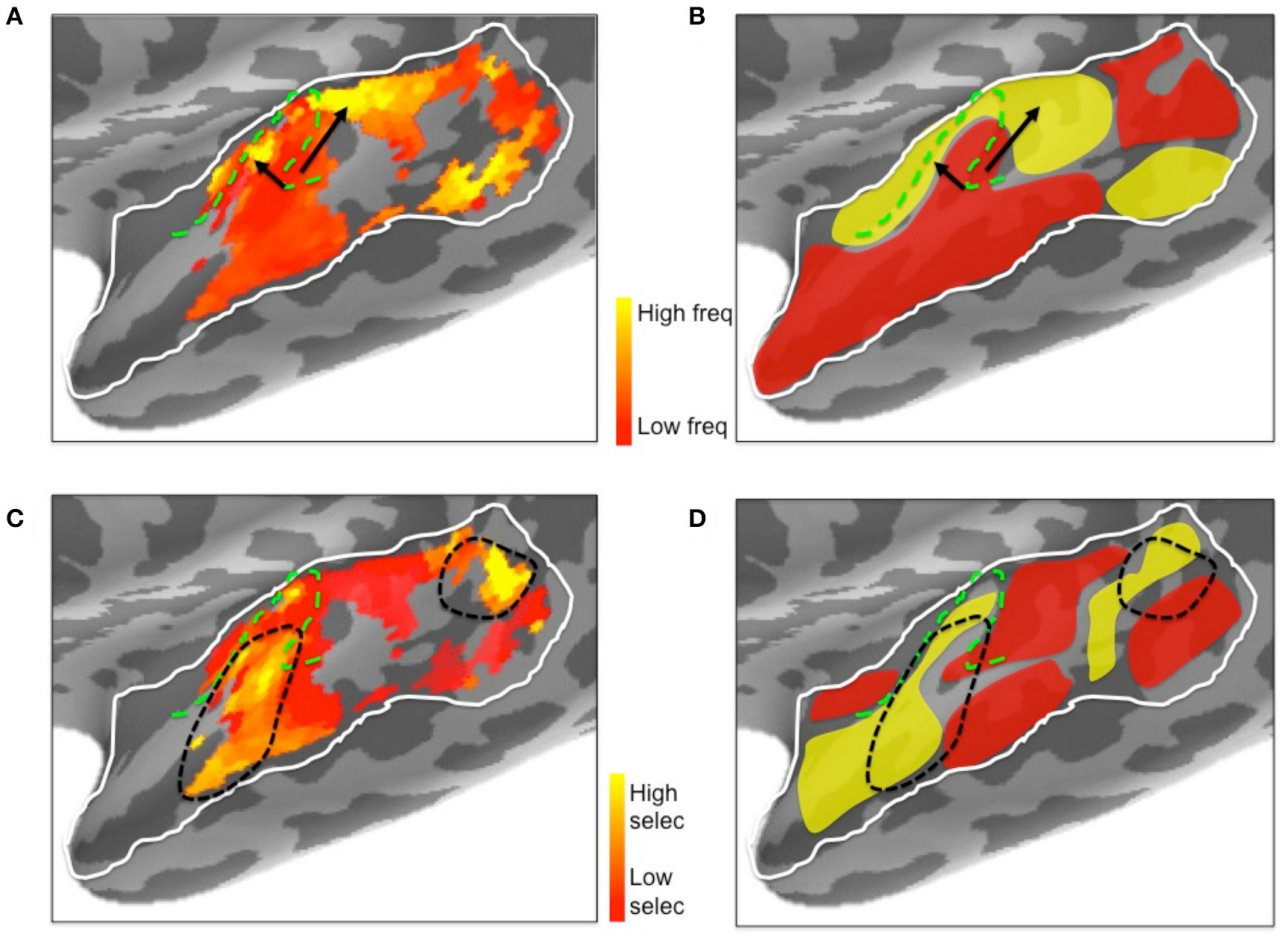
**Illustrative comparison of the ssRSA/EMEG results (A,C) with existing left hemisphere tonotopic maps based on fMRI techniques (B,D). (A)** Frequency preferences mapped from EMEG and **(B)** from fMRI. Black arrows indicate the directions of suggested low to high frequency gradients in V-shaped orientation. **(C)** Frequency selectivity mapped from EMEG and **(D)** from fMRI. Black dashed lines indicate higher frequency selective regions in and near primary auditory cortex. The schematic fMRI-based diagrams **(B,D)** were generated based on information from Saenz and Langers (2014) and Moerel et al. (2014). Dashed green lines show the outlines of Heschl's gyrus.

Figure 10 summarizes some salient aspects of the relationship between the ssRSA/EMEG results and current tonotopic maps based (primarily) on high field (3 T/7 T) fMRI, as summarized in recent reviews and commentaries (e.g., Baumann et al., 2013; Moerel et al., 2014; Saenz and Langers, 2014). As these reviews make clear, there has been a surprising degree of controversy over the past decade about the exact tonotopic organization of the auditory “core” in human auditory cortex, reflecting both the relatively small size and inaccessibility of the relevant brain areas and the rapid evolution of neuroimaging technologies providing a succession of new perspectives on neural responses in these regions.

Research in non-human primates has long converged (e.g., Merzenich and Brugge, 1973) on the view that mirror-symmetric tonotopic gradients are found in the AI and R region of core auditory cortex in the macaque. These frequency preference gradients, running from high to low to high, were generally agreed to fall along the posterior-anterior axis of the macaque auditory core, either collinearly (e.g., Kaas and Hackett, 2000) or forming an angled pattern converging at the AI/R midline (e.g., Baumann et al., 2010). It has been less clear how (and whether) these core regions, and their tonotopic gradients, map onto human primary auditory cortex, and onto Heschl's gyrus in particular. Earlier MEG studies (e.g., Pantev et al., 1995), using less accurate dipole-based analysis methods, were interpreted as consistent with a simpler tonotopic arrangement in humans, with a single gradient running low to high, lateral to medial along Heschl's gyrus. Only with the advent of high field fMRI studies (e.g., Formisano et al., 2003) did it become clear that human auditory cortex also exhibited multiple mirror-symmetric high-low-high tonotopic gradients akin to those seen in macaque. Even so, there has been continuing disagreement about the orientation of these gradients relative to Heschl's gyrus, with the collinear arrangement suggested by Formisano et al. (2003), running high-low-high along the gyrus, being challenged by Humphries et al. (2010) and others, arguing that tonotopic gradients run perpendicularly across Heschl's gyrus rather than along it.

Recent reviews by Baumann et al. (2013) and subsequently Saenz and Langers (2014), argue convincingly for a third view of the orientation of symmetric tonotopic gradients in and around Heschl's gyrus, related to the angled orientation proposed in earlier macaque studies (e.g., Baumann et al., 2010). On this account, there is a clear low-frequency trough in the mid-to-lateral half of HG, which is flanked by high-frequency representations, running anteromedially toward the planum polare and posteromedially toward the planum temporale (see Figure 2 in Saenz and Langers, 2014). Both Baumann et al. (2013) and Saenz and Langers (2014) argue that this V-shaped arrangement of the tonotopy gradient also has the advantage of being much closer to current views of macaque auditory cortex.

Inspection of the current ssRSA results (Figure 10) suggests a very similar arrangement. Figure 10A (reproducing the LH frequency preference map from Figure 7) shows a predominance of lower frequency preferences in Heschl's gyrus. Higher frequency regions are located anteromedially and posteromedially, forming a potentially V-shaped organization of symmetric high-low-high tonotopic gradients. This arrangement, indicated by black arrows in Figure 10A, corresponds well to the schematic diagram (Figure 10B) of tonotopic maps for the same region derived from recent high-field fMRI studies (Moerel et al., 2014; Saenz and Langers, 2014). It is also consistent with the layout described by Moerel et al. (2012), based like the current study on frequency preferences elicited from natural sounds.

Outside Heschl's gyrus and surrounding areas (likely corresponding to core and belt auditory regions), both ssRSA and fMRI schematic maps exhibit a predominant LH preference for lower frequencies, both in middle and anterior STG and STS, and in posterior STS. This may reflect a tuning of these areas to the frequency properties of speech in particular (Moerel et al., 2012; Norman-Haignere et al., 2013). In the current study we explored a wide frequency range from 30 to 8000 Hz. While we found clear evidence of (peak) frequency sensitivity up to 8000 Hz (see Figure 8), the (center) frequency preference distributions (Figures 6A,B) suggest both auditory cortex and surrounding superior temporal regions were predominantly driven by the lower end of this frequency range, from 200 to 2000 Hz.

Frequency selectivity, referring here to the width of the Gaussian tuning curve computed at each spatiotemporal searchlight window, has featured prominently in neurophysiological studies of the response properties of individual neurons (Kanold et al., 2014). In the non-invasive human literature, where any tuning curve will average over many thousands of neurons with potentially heterogeneous frequency selectivities, less emphasis has been placed on this aspect of the neural substrate for auditory cortex. An exception is the recent fMRI research by Moerel et al. (2012, 2013), who also computed Gaussian tuning curves from which selectivity can be derived. Their results, schematically represented in Figure 10D, show substantial similarity with the results we obtained. The ssRSA map of the SD of the tuning curve (Figure 10C) shows a LH region of narrower frequency selectivity (marked by black dashed lines) in the anterolateral half of Heschl's gyrus, extending into anterior STS and STG, with a second well-defined patch of narrow selectivity in posterior STS. These are both regions that also show frequency selectivity in the fMRI studies (Figure 10D).

The ssRSA frequency selectivity results for Heschl's gyrus, both on the left and on the right (see Figure 9) can also be linked to neurophysiological research with non-human primates. This research uses several methods to distinguish core from belt auditory cortex, including a functional definition based on the width of the frequency-tuning curve. Neurons in the core auditory field have much sharper tuning curves compared with neurons in the belt region (Rauschecker et al., 1995; Hackett et al., 1998). The results here are consistent with this, showing that the primary auditory cortex located midway in Heschl's gyrus was more frequency selective than the areas surrounding it.

### Methodological implications

The novel combination of techniques presented here has three characteristics which, taken together, distinguish this research from previous studies of tonotopy—and, indeed, of cortical function more generally.

First, the analyses are conducted in MRI-constrained EMEG source space, using minimum norm distributed source reconstruction methods, which map signals recorded at the scalp back to the whole brain cortical surface (defined as the white matter/gray matter boundary). While necessarily noisy and imperfect, these are nonetheless the best available non-invasive methods for measuring and representing the dynamic electrophysiological events that underpin real-time brain function, with millisecond-level temporal resolution and potentially sub-centimeter spatial resolution.

Second, the ssRSA method provides a statistically unbiased and robust means of interrogating this representation of real-time neural activity in order to determine the *qualitative* functional properties of the neural computations supported by this dynamic electrophysiological activity in EMEG source space. Every model RDM encodes (implicitly or explicitly) a theoretical claim about the functional dimensions that constrain neural activity within the spatiotemporal window sampled by the ssRSA searchlight procedure. The match between model RDM and brain data RDM is not simply evidence that a pattern match can be found (as in machine learning-based pattern-classification techniques) but that this is a pattern match with specific neurocomputational implications.

In the current study, the model RDMs express a neurobiologically plausible theoretical model of how frequency variation is encoded in the early stages of auditory processing, modulated by the spectral properties of a given auditory input dynamically changing over time. The significant fit of these multiple model RDMs to the correlational structure encoded in each brain data RDM therefore licenses direct inferences about the qualitative properties of the neural computations being conducted within the spatiotemporal window covered by that data RDM—in this study, inferences about the frequency preferences of the brain area being sampled, and the selectivity of these preferences. Critically, these inferences are not based on simple variations in the amount of activity associated with a given frequency dimensions, but rather on the multivariate correlational pattern elicited (in this case) across a 400 × 400 stimulus matrix.

Thirdly, the ssRSA approach (and RSA in general) allows the use of naturalistic stimuli—in this experiment naturally spoken words—that correspond more closely to the kinds of sensory inputs to which the neural systems of interest are normally exposed. Naturally spoken words present the auditory object processing system with frequency variation in its natural environment—i.e., reflecting the complex mixture of spectral patterns imposed on the speech output by the human speech apparatus in order to serve specific human communicative functions. This is the ecologically central environment for human auditory processing of spectral variation, and a necessary context in which to study these processes. Studies using artificially generated tone sequences, in a psychophysical testing format, may pick out neural response properties that are not in fact the salient modes of processing in more ecologically natural contexts^3^.

The ability of ssRSA to use naturalistic stimuli derives from its use of model RDMs. In so far as the relevant dimensions for constructing the correlational structure of a model RDM can be extracted from a given stimulus set—in this study frequency variation from spoken words—then any stimulus set which allows this is potentially usable. This also means that multiple functional dimensions can be extracted from the same stimulus sets. Naturally spoken words exhibit a rich set of properties over many dimensions, ranging from the acoustic to the phonemic to the lexical. ssRSA allows us to probe the neural responses to such stimuli across any functional dimension for which it is possible to specify a model RDM—for an example of a preliminary study using lexical models to probe word-recognition processes in the same set of words (see Su et al., 2012). Here we demonstrate this for the salient dimension of frequency-based processes, especially critical for speech comprehension. In complementary research on the same stimulus set, we can interrogate EMEG source space along phonetic and phonemic dimensions, providing a broader functional context for interpreting the auditory processing characteristics observed in the current study of tonotopy.

## Conclusions

In summary, the current results present a credible and realistic analysis of the neural distribution of frequency sensitive processes in human bilateral superior temporal cortex. Frequency preferences in and around Heschl's gyrus are consistent with current proposals for the organization of tonotopic gradients in primary acoustic cortex, while the distribution of narrow frequency selectivity similarly matches results from the fMRI literature. While we cannot provide exact measures of localizational accuracy in the spatial domain, the group level maps provided here seem comparable to those that have emerged from fMRI or ECOG studies, and a considerable advance over earlier MEG research.

More generally, the *in-vivo* and non-invasive ssRSA approach can be combined with both neurophysiological and cytoarchitectonic methods for locating and subdividing auditory cortex. For example, when measuring the neural response in human auditory cortex using single-unit recording in selected patients, frequency preference and selectivity can be mapped using techniques directly comparable to those used in non-human primates. New quantitative MRI techniques allow the mapping of tissue microstructure and provides information about density of myelination of the neurons, resulting in an *in-vivo* map of human primary auditory cortex (Dick et al., 2012). We believe that combining advances in imaging techniques (fMRI, EEG, and MEG) with advanced computational methods such as ssRSA will provide important new opportunities to unravel the functional organization of human auditory cortex.

### Conflict of interest statement

The authors declare that the research was conducted in the absence of any commercial or financial relationships that could be construed as a potential conflict of interest.

## Footnotes

^1^The output of this sound transduction system is essentially flat up to 5 KHz with a 12 dB drop off at 6 KHz.

^2^The question of whether the tonotopic organization of auditory cortex may have dynamic properties, and therefore vary over time (possibly as a function of the distribution of recent inputs), is an interesting one, but outside the scope of this report.

^3^It is worth noting, nonetheless, that Moerel et al. (2012), who used both natural words and pure tone sequences, found broadly similar spatial maps of frequency sensitivity for both types of input.
